# Breastfeeding Experience During COVID-19 Pandemic in Indonesia: Strengthening and Weakening Elements

**DOI:** 10.21315/mjms2022.29.3.11

**Published:** 2022-06-28

**Authors:** Giyawati Yulilania Okinarum, Wahyu Rochdiat

**Affiliations:** 1 Department of Midwifery Science, Faculty of Health Science, Universitas Respati Yogyakarta, Yogyakarta, Indonesia; 2 Department of Nursing Science, Faculty of Health Science, Universitas Respati Yogyakarta, Yogyakarta, Indonesia

**Keywords:** COVID-19 pandemic, elements, breastfeeding, strengthening, weakening

## Abstract

**Background:**

Indonesia is severely affected by the COVID-19 pandemic, particulary in the context of exclusive breastfeeding. Breastfeeding is the safest for babies in disaster situations, and the success of breastfeeding is a social and collective responsibility, as well as also a woman’s decision. The focus of this study is to explore the strengthening and weakening elements of breastfeeding experience during the COVID-19 pandemic.

**Methods:**

This is an exploratory qualitative study design, conducted in Yogyakarta Special Region Province, with nine lactating mothers who participated in the study. Interviews were audio-recorded, transcribed, translated and thematically analysed.

**Results:**

Strengthening elements in breastfeeding mothers during the pandemic were maternal affection to her baby, support system from family and community, and having adaptive coping strategy. The weakening elements were impaired comfort, insufficient milk supply, financial problem, parenting problem and indifferent husband.

**Conclusion:**

Breastfeeding a baby is appropriate even in a pandemic situation. Thus, to achieve good clinical practices that encourage breastfeeding, the support process should include a subjective and social component, and strengthen the support network of breastfeeding mothers in this COVID-19 pandemic situation. This will help to resolve the barrier in breastfeeding and the babies will receive their rights.

## Introduction

Indonesia is one of the countries affected by the COVID-19 pandemic. People are often deprived of resources to survive due to natural disasters. These natural disasters can lead to sporadic increases in unemployment and lack of food, making situations worse. Breastfeeding will continue to provide the cleanest and safest nutrition for babies and serve as a normative standard for infant nutrition in disaster situations (1). Most studies documented no respiratory or other illness in neonates born to mothers with confirmed/suspected infection with SARS-CoV-2 (2–3). In addition, a recent report found that 12 out of 15 mothers (80%) previously infected with COVID-19 had a strong IgA SARS-CoV-2 immune response in breast milk (4).

Recent research estimate that the increase in exclusive breastfeeding in the world can prevent 823,000 child mortalities and 20,000 maternal mortalities from breast cancer annually. The benefits of breastfeeding have been expanded due to the epidemiological and biological discoveries over the past decades (5). Despite this, low rates of breastfeeding are still reported in Indonesia. The prevalence of exclusive breastfeeding in children under 6 months old was 54.3%, less than 80% which is the national target of exclusive breastfeeding coverage (6). In Indonesia, the proportion of mothers who did not breastfeed their babies because of lack of milk supply was 65.7%, and 68.3% babies in the 0–6-month-old of age group were not breastfed for the same reason (7).

Policies and strategies developed in Indonesia to promote, protect and support breastfeeding are considered limited and fragmented. So, it is necessary to increase the legal status of existing breastfeeding policies to improve the commitment towards increased use of breast milk (8). During the COVID-19 pandemic, the government has superficially explored the roles of families, health professionals, communities and the state in the context of supporting breastfeeding. The success of breastfeeding is a social responsibility collectively, besides being the decision of the woman. A study reported that maternal intentions, maternal and child health conditions, maternal occupation, family support, health professionals and cultural issues influenced the incidence and duration of breastfeeding (9). The study reported the importance of more studies that seek to develop maternal and professional skills for sustaining breastfeeding and strengthening strategic actions to support breastfeeding mothers (10).

Research that examines the biological dimension of breastfeeding can impact strengthening the social role of breastfeeding during the COVID-19 pandemic. It is necessary to ask the question: “What are the elements that strengthen and weaken breastfeeding in lactating women’s perspective who are affected by the pandemic?” To answer that question, we adopted this study’s aim, i.e. to explore breastfeeding experience, such as the strengthening and weakening elements in breastfeeding mothers during the COVID-19 pandemic.

## Methods

We used an exploratory qualitative design with purposive sampling techniques to recruit breastfeeding mothers who had mild and moderate stress based on the Perceived Stress Scale-10 (PSS-10) instrument. A qualitative design approach provides a comprehensive understanding of a particular situation, a description that conveys participants’ experiences or actions from their perspective (11). Qualitative approaches are the basis for qualitative research and include meaningful methodological approaches without a theoretical framework (12). In addition, the qualitative findings of exploration can provide information about new interventions in the socio-cultural context of the participants (13). This exploratory qualitative study is considered appropriate because the comprehensive study aims to gain an in-depth understanding of the paradigm seen from breastfeeding mothers who have experienced breastfeeding during the COVID-19 pandemic.

### Study Settings and Participants

The study was conducted in Yogyakarta Special Region Province, Indonesia. Breastfeeding mothers, who were impacted by the COVID-19 pandemic, either psychologically or physically and did not breastfeed their baby at the time of data collection in different districts in the province, participated in the study.

### Participants’ recruitment

Postpartum and lactating mothers (< 3 months) were purposively recruited from four districts in the urban setting of Yogyakarta Special Region Province. Lactating mothers who did not breastfeed their baby during data collection were not included in the study. Eligible participants were invited to participate in the study by the author/researcher. They were informed about the aims of the study and the procedure of data collection. Two of nine participants were victims of sexual violence (i.e. rape) before they had a baby and both of them did not marry.

### Data collection

All authors (GYO and WRM) collected and analysed the data. In total, nine interviews were completed and generated. The required number of participants was not determined before the study was carried out. The researchers collected data until it reached a saturation level, i.e. when new data no longer appeared. The purposive sampling method was used because of the difficulty in identifying participants. Face-to-face interviews were conducted using topic guidelines and open-ended questions about the experience of breastfeeding during the COVID-19 pandemic. The interviews explored the following topics: what was it like to have a baby in a COVID-19 situation, how did they feel about the impact of COVID-19, the changes that had occurred in their lives since the initial period of the pandemic, their plans for breastfeeding during the pandemic, from whom and where they would find any help, as well as strengthening and weakening factors during breastfeeding in a pandemic situation.

Data were collected between November and December 2020 and each interview lasted approximately 45 min–60 min. All interviews were audio-recorded and transcribed word-by-word (verbatim) in Javanese and translated into Indonesian by the lead researcher. Another person confirmed accuracy between the audio-recording and the transcription as an independent assessor, in which case a confidentiality agreement was made. The nine transcripts were translated into English for data analysis. The independent bilingual assessor was tasked with ensuring the meaning of the narrative and the accuracy of the translation into English and back-translation into Indonesian. He also confirmed the interpretation of statements and phrases that could not be directly translated between languages.

Informed consent was explained to each participant before the interview was conducted. The interviews were located in a place where the maximum privacy of participants could be guaranteed, i.e. in each participant’s house. Confidentiality of information was strictly maintained by research ethics. Before each interview session began, the researcher allocated sufficient time to build trust and good relations with the participants. After the interview was completed, a cash receipt of IDR50,000 was presented.

### Data Analysis

Data were analysed using six stages of thematic analysis: i) data introduction; ii) initial codes generation; iii) theme search; iv) theme study; v) theme definition and naming, and vi) reporting (14). NVivo 12 facilitates researchers to perform iterative data management and emerging themes were arranged according to the semantic content of the codes (15). Throughout the study conduct and analysis process, a reflexive approach allowed the lead researcher (GYO) to think about and discuss potential personal effects from her own biases (16). Interview recordings were transcribed word-by-word (verbatim) and examined by a second researcher (WRM). GYO and WRM re-read all transcripts and built a theme coding framework using the constant comparison method, in which new data were compared to previously collected data to refine the ‘labels’ assigned to themes in the data. Then, analytic themes were discussed and further developed by the primary researchers (GYO and WRM).

## Results

At the time of data collection, all participants in this study were still breastfeeding. The majority of participants had a higher education but did not work and earned from their husbands slightly more than the provincial minimum wage. There were only two unmarried participants (Table 1). One participant was a rape victim, while the other was the result of incest with her brother. According to the findings of the interviews, there are a variety of factors that strengthen and weaken participants’ breastfeeding during the COVID-19 pandemic (Table 2).

### Strengthening Elements

#### Theme 1: Maternal affection to her baby

The reasons to keep breastfeeding and preventing COVID-19 transmission by these mothers having a baby are as follows:

“I have a baby, so I did not do the things that can put my baby and my family in a dangerous situation.” (P5)

“If breastfeeding mothers can be vaccinated, I am the first one who would like to register. Because I have a baby and I must protect her.” (P8)

#### Theme 2: Support system from family and community

Since the pandemic began, most activities have been carried out online. It also affects breastfeeding mothers’ behaviour in seeking information to increase knowledge by taking online classes and utilising live features on social media, such as Instagram live and Facebook live.

“...so I can join on IG live and free online class, it can reduce my stress because of this pandemic.” (P3)

Support from husband and parents also could reduce stress and support breastfeeding in a pandemic situation.

“Even though my husband and I live separately, but he absolutely supports me for exclusive breastfeeding.” (P5)

“My family are my support systems. My parents always help me when I need them to take care of my baby.” (P5)

“I almost committed suicide, but my parents strengthened me.” (P7)

“My mother told me to clean my breast and nipple, every time I took a bath. Thank God, my mother always supports me.” (P9)

Another support system from online community could build the stress relief in breastfeeding mothers.

“I got support to keep breastfeeding my baby from my community on Facebook.” (P1)

#### Theme 3: Having adaptive coping strategy

This study found one of the coping strategies carried out by breastfeeding mothers to reduce stress in lactating during a COVID-19. This strategy is emotion-focused coping as a strategy for reducing the stress-causing situation while breastfeeding during the pandemic through positive reappraisal, distancing, and escape.

“At the end of the day, I thought that God would be with us and provide sustenance for us, even in this pandemic situation.” (P4)

“I’m grateful for this pandemic situation, with the government policy to stay at home and keep the distance. Thus, I do not interact with my neighbours who mean to me.” (P7)

“I’m trying to forget the bad things that happened in my past. I do not follow bad people on my social media. I try to focus on selling, keep myself busy, shalat and several times I participated in online recitation.” (P7)

Problem-focused coping by seeking social support is also a coping strategy for reducing and healing stress.

“My friends in breastfeeding community or perinatal mental health community on Facebook always help me to give any information about healthy life so that it can make me better and I am not confused anymore.” (P3)

### Weakening Elements

#### Theme 4: Impaired comfort

The women in this study reported that they experienced some uncomfortable situations, either physical or emotional, that detained the breastfeeding process. This situation includes nipple pain, nausea, vomiting, dizziness (physical), fluctuating mood, panic attacks and being frustrated all the time (emotional).

“I was stressed in early breastfeeding. I kept crying. I got nipple pain. Nevertheless, I did not stop breastfeeding for my baby.” (P8)

“My first son is already in Grade 3 of elementary school. So, no wonder if I forget how to breastfeed my baby. However, I keep breastfeeding even though low milk supply.” (P8)

“I was getting dizzy. How is my husband’s life in Jakarta? Is he ok? Because the COVID-19 transmission in Jakarta is the highest one in this country”. (P5)

“I got nausea, vomiting and dizziness because of this pandemic.” (P7)

“Now I am jobless. My moods are fluctuative and not stable.” (P6)

“I often get angry because I am too afraid if my baby and I get the COVID-19 transmission. Yesterday my family came to our home to visit my baby, I wanted to tell them to go back home but I could not. Because they came here from another city. I am very worried and afraid because of this COVID-19 transmission!” (P1)

“A week ago, I was getting cough and panic attacks after my family visited my baby. Fortunately, it just a mild influenza.” (P1)

#### Theme 5: Insufficient milk supply

This study found that breastfeeding mothers who are stressed due to being affected with COVID-19 have insufficient milk supply.

“I often get angry, then suddenly I have a low milk supply, and my baby is crying all the time.” (P2)

“Maybe because I stressed, the milk supply be on the wane. I pumped my breast, and I only got 10cc of breast milk.” (P5)

“...I was stressed in the beginning of a pandemic. It makes my milk supply are low.” (P8)

“...I keep breastfeeding even though low milk supply.” (P8)

#### Theme 6: Financial problem

The same concern was voiced among women whose husbands had less pay and were laid off: financial problem. Their primary concerns were for their life and the future of their children.

“Looking for a job is so hard during this pandemic situation. It makes my milk supply low, and you know... the father of my baby is in a prison, he did not want to be a responsible person as a man. I can survive without him.” (P7)

Instead, these women kept breastfeeding because formula milk was not affordable for them.

“I did not breastfeed my first son. But now, I have to breastfeed my baby. A formula milk is too expensive for us in this situation of a pandemic.” (P2)

#### Theme 7: Parenting problem

It was observed that most of the participants in this study experienced some form of parenting problem.

“I often get angry, then suddenly I have a low milk supply, and my baby is crying all the time. My first son has to attend an online school. His teacher did not teach my son, but we have to pay tuition fees.” (P2)

“This pandemic situation is tough for me, a young single parent mom. She is my first daughter, I love her, even though she was born because of an ‘accident’.... an unwanted pregnancy.” (P7)

Most of the women in the study had to shoulder the responsibility of caring for the baby and cleaning the house simultaneously. Meanwhile, their husband did not take a role in household chores.

“My husband felt tired after work, so he did not help me when he was already at home. I did all the things at home, including taking care of my baby and cleaning the house at the same time.” (P2)

#### Theme 8: Indifferent husband

Mothers who did not get breastfeeding support from their partners made them skeptical about their milk supply. It is essential that the father as the woman’s partner support positively and to actively develops maternal confidence in breastfeeding, a known predictor for maintaining breastfeeding.

“I only live with my husband, and he does not support me to breastfeed my baby. He always asked me to stop breastfeeding and start feeding the baby with formula milk. He felt that my baby was crying all the time because she was starving and my low milk supply. He bought formula milk, at that time, but I did not give it to my baby. Even though, actually, I became doubtful about my milk supply because of my husband’s attitude.” (P1)

“...My husband didn’t help me like clean the house or anything else.” (P2)

“...My husband gives me a support for exclusive breastfeeding, but no action, talk only, like ‘I support you,’ that is all.” (P3)

## Discussion

The perception of breastfeeding is a construction knowledge coming from the senses and memories that vary depending on the information sources, social conditions, culture, economy, emotions, beliefs, skills, objectives and needs (17). A study conducted on the population of Mexican women in the United States identified that cultural burdens are fundamental to women’s decision to breastfeed (18). Breastfeeding’s cultural baggage as weakening and strengthening in lactating will undoubtedly be a different consideration during the current COVID-19 pandemic. Our findings showed that women in the Special Region of Yogyakarta had strengthening and weakening elements in breastfeeding during the COVID-19 pandemic.

### Strengthening Elements in Breastfeeding Mothers During the Pandemic

#### Maternal affection to her baby

Breast milk is an excellent source of nutrition for newborns and infants (19). It protects against pneumonia, diarrhoea, gastroenteritis, respiratory infections, obesity, type 1 and type 2 diabetes, asthma and other illnesses (20). According to a recent study, 12 of 15 mothers (80%) who had previously been infected with COVID-19 had a strong IgA SARS-CoV-2 immune response in their breast milk (4).

Since they were aware of the benefits of breastfeeding, the mothers in this study believed they should breastfeed their children. This decision is based on the mother’s love for the child she has given birth to after a long and challenging labour. These findings were consistent with a study in Portugal that found more affection in women who received support from breastfeeding counseling groups (21). Breastfeeding is also important from an emotional standpoint in the mother-baby interaction because of the cooperation, closeness and visual contact, allowing reciprocity and the formation of gradual bonds, i.e. a rich and complex bond between a mother and her child.

The majority of participants have a high level of education, which makes it easier for them to understand the benefits of breastfeeding in protecting their children during the COVID-19 pandemic, resulting in a stronger attachment to babies. These findings were consistent with a study in Portugal which found that women with low literacy levels have lower levels of maternal affection. Pinto et al. (21) showed that the level of education affects breastfeeding motivation due to more access to information. Maternal affection was not influenced by age, marital status, employment status, residence, number of pregnancies, pregnancy monitoring, length of breastfeeding, time interval before breastfeeding, pregnancy plans, desire to become pregnant or type of delivery.

A survey of neonates in Pomerania (SNiP-study) reported that women with no motivation to provide exclusive breastfeeding had lower education and lower average incomes (22). This study found the same results as another study because women with a high education level were more likely to continue breastfeeding to prevent SARS CoV-2 transmission. We also discovered that they continued to breastfeed their child despite having a low income because their husbands received less pay as an impact of the pandemic situation. They felt that formula milk was not affordable for them.

#### Support system from family and community

Our findings showed that unconditional support from husbands, families and communities immensely helped these women maintain their commitment to breastfeeding during this pandemic. This finding was consistent with other studies in Nepal that mothers who gained support from their husbands were 10 times more likely to breastfeed (23). Husbands’ verbal support and active participation in breastfeeding activities makes mothers feel more capable and confident about breastfeeding (24). Husbands are highly influential in influencing mothers’ decisions about breastfeeding their children and whether or not to continue breastfeeding (25). According to a Nepalese study, mothers who lived in extended families were more likely to practice exclusive breastfeeding, owing to better family support (26). Thus, community and family support, particularly from the husband, is critical for successful breastfeeding practice and exclusive breastfeeding for up to six months.

#### Having adaptive coping

All of the participants faced some problems related to multiple problems in the pandemic situation. They realised that becoming a breastfeeding mother during the pandemic was very challenging and prone to stress. To overcome that, our participants used two types of coping strategy. The first one is a problem-focused coping by seeking social support such as joining on Instagram live or Facebook live, webinars and talking with their friends in the breastfeeding community on Facebook. The second one is emotion-focused coping through positive reappraisal being grateful and surrendering to God, following physical distancing during a pandemic and escaping from negative people who remind them to bad experiences in the past, such as in the case of a victim of sexual violence.

According to Lazarus’s theory in Biggs et al. (27), coping has two main functions. It can change the problems causing stress or it can regulate an emotional response to the problem. Based on this theory, coping can be divided into two types: i) emotion-focused coping, the goal is to control emotional responses to stressful situations. People can regulate their emotional responses through cognitive and behavioural approaches, and ii) problem-focused coping which aims to reduce the demands of a stressful situation or expend resources to deal with it.

However, all participants attempted to handle all the problems by using coping strategies. Participants performed coping strategies not only after the problems appeared but also before such problems appeared as they did anticipatory and preventive coping so that coping can be seen as something that can be done before the stress or distress emerges (28).

Anticipatory coping means that someone shows an effort to deal with an imminent threat or even a critical event shortly. Efforts are made to build future resources or minimise the severity of the impact of potential distresses through preventive coping (29).

Besides taking the anticipatory and preventive coping, participants also performed reactive coping, which means to deal with an ongoing stressful encounter or one that has already happened (29). After the problem appeared, participants tried to face it by using coping strategies such as problems and emotion-focused coping that can be concluded as reactive coping. According to Lazarus and Folkman’s theory in Ragin (30), coping is defined as cognitive or behavioural actions to manage when situational demand exceeds someone’s resources.

### Weakening Elements in Breastfeeding Mothers during the Pandemic

#### Impaired comfort

A common reaction to the COVID-19 pandemic is stress. The COVID-19 outbreak, which occurred unexpectedly, resulted in significant changes in people’s lives. These changes create uncertainty about the future, which is a stressor that eventually leads to stress (31). Nursing mothers are also vulnerable to stress as a result of the COVID-19 pandemic. The severity of the symptoms of this disease causes postpartum mothers to be concerned about infecting both the baby and themselves.

Physiological discomfort can also trigger the mother to discontinue breastfeeding (32–33). According to the findings of this study, breastfeeding mothers face challenges such as nipple pain, nausea, vomiting and dizziness. These findings were consistent with a study in Qatar, which found that nipple disruption is one of the reasons mothers stop breastfeeding (34). The pain felt when the baby sucks the milk causes stress in the mother. This condition is due to increased cortisol, which plays an essential role in stress (33, 35).

Other physical discomforts such as nausea, vomiting and dizziness can trigger processes in the brain that decrease milk production and cause emotional reactions that make the mother reluctant to breastfeed. Physical discomfort usually involves the hormone serotonin. This hormone can also have an impact on the mother’s mood and they become sad and unmotivated to breastfeed their children due to a disruption in the serotonin hormone balance, which was initially triggered by physical discomfort (35–36).

#### Insufficient milk supply

The perceptions of insufficient milk supply would reinforce a lack of confidence in the mothers’ milk supply. Breastfeeding mothers have the perception that they are not producing enough milk for their babies. Some of the things that make mothers have this perception are because babies seem never satisfied after being breastfed, so mothers often cry and experience stress. This finding is in line with research in Western Australia that, at three weeks after birth, 44% of mothers have problems regarding the perception of insufficient milk supply. There is a need for professional guidance immediately after birth (37).

Adequate milk drainage from the breast is critical, especially if the goal is to increase milk production. Proper attachment and positioning of the baby is required to optimise effective drainage. One recommendation for facilitating effective drainage is to make the mother as physically and psychologically comfortable as possible. In this context, the expected psychological condition is that the mother is not stressed and in a calm condition (38).

#### Financial problem

The findings of this study indicate that economic factors are the main factors that increase stress during the breastfeeding process. Many people have lost their jobs due to COVID-19 or have had their income reduced if they are still fortunate to be working. Many people’s socioeconomic status has changed dramatically due to the pandemic (39–40). To combat the virus’s spread, the government has made it mandatory to enforce strict health protocols that limit people who leave their house. As a result, business owners who rely on customer purchases reduce salaries or even reduce the number of employees.

Since their husbands’ income decreased during the pandemic, participants felt a change in socioeconomic status. Participants in this condition are concerned about their child’s uncertain future as a result of COVID-19. This feeling exacerbates the stress they experience while breastfeeding, resulting in low milk supply. This finding is consistent with previous research, which found that low income causes an increase in perceived stress in breastfeeding mothers, resulting in a decrease in their self-confidence to breastfeed (41). Without economic certainty, the mother’s psychological state will be disrupted, resulting in the interruption of the breastfeeding process (39, 41).

#### Parenting problem

Another interesting finding from this study is that one of the participants complained about her husband’s irresponsibility, who had to go to prison for committing a crime. Since she did not have a job to provide economic security, the participant’s stress level increased. This stressor reduced her milk supply. One of the factors that predicts the success of exclusive breastfeeding is social support and one of the most critical social supports comes from the husbands of breastfeeding mothers (33).

The parenting role includes activities and attitudes toward the baby and includes the roles that both parents fill in the family as a whole (42). In this case, the fathers focus on working amid the pandemic without paying attention to the mothers who needs help in caring for her child and support during breastfeeding. Fathers seem to be having a hard time understanding that they have become a father and are too focused on their other responsibilities to earn a living amidst the hardships of the COVID-19 pandemic.

Most parents stated that their focus on working with their partners was crucial. They helped each other (42). Participants in the study also reported feeling pressured to fulfil their parenting roles perfectly and many mothers reported a lack of coping because of the enormous demands they felt, one of which was the changes in life during the pandemic. Exploring this topic further shows that parents’ lack of coping is primarily due to their own emotions and demands, especially during the COVID-19 pandemic. Most of the mothers in this study research had very high expectations of themselves and were taught that parents should always be the prominent people around their babies.

#### Indifferent husband

When mothers see their partner’s support in the form of verbal encouragement and active participation in breastfeeding activities, they feel more capable and confident in their ability to breastfeed. Mothers who have partners who appear ambivalent, are motivated solely by ‘what is best for the baby,’ or provide negative feedback about breastfeeding have lower confidence in their ability to breastfeed (24).

Our study reports that mothers desperately need help from their husbands during breastfeeding. The help needed is not only verbal but also non-verbal, which is shown by concrete actions. More specifically, in previous studies, fathers who actively supported and encouraged mothers by helping position babies and carrying snacks and diapers, strongly influenced mothers’ perceptions of trust in breastfeeding (24).

According to some research study, mothers who reported negative support from their partners had lower self-confidence in milk production and lower self-efficacy in breastfeeding as a measured by BSES. Previous research has found that a woman’s self-confidence in breastfeeding can be influenced by her partner (43–45).

## Limitations

Considering the subject’s sensitivity experiencing moderate stress in two out of nine participants who were subjected to sexual violence before childbirth, it can be assumed that some bias may have come into play during the study. This limitation is overcome by establishing good relationships between participants and researchers. This study was unable to follow breastfeeding mothers’ lives to see if lactation behaviour was still practiced until at least six months to achieve exclusive breastfeeding even during the COVID-19 pandemic with the strengthening and weakening elements that still exist. Moreover, it was conducted in an urban setting with characteristics that may be different from rural settings. Thus, in future studies, it is better to expand the coverage.

## Conclusion

Breast milk is food with complete and natural nutrition that babies need. In addition, it contains other elements that promote child growth and protection against infection, including the transmission of SARS-CoV-2; breast milk also contains a strong IgA SARS-CoV-2 immune response. Apart from being caused by the mother’s immune defenses to the child, the possibility of breast milk being contaminated is also low. It will provide allergy protection and adaptation to other foods.

Breastfeeding is also important from an emotional standpoint in the mother-baby interaction because of the cooperation, closeness, and visual contact, allowing reciprocity and the formation of gradual bonds, i.e. a rich and complex mother-child bond.

It can be concluded from this qualitative inquiry that breastfeeding mothers had strengthening and weakening elements in lactating during the COVID-19 pandemic. These women cited valid reasons for their strength in lactating during the pandemic, such as maternal affection to her baby, support system from family and community, and adaptive coping strategy. These reasons point to the ability of these women to keep breastfeeding during the pandemic. Other findings highlight the weakening elements in breastfeeding such as impaired comfort, insufficient milk supply, financial problem, parenting problem and indifferent husband. Although these women had a weakening element in breastfeeding during the pandemic, these circumstances led them to keep lactating for their babies, they made any efforts to avoid problems to keep breastfeeding during the pandemic from a different perspective. Uncertainty in monthly income because of salary deduction prompted them to continue breastfeeding and not buy formula milk because the latter was not affordable for them. As a result, these women adopted a more empowered position and regained control of their circumstances, even though it was difficult for them.

## Figures and Tables

**Table 1:** Baseline characteristics of participants (*n* = 9)

Characteristics		*N* (%) or mean (SD)	Median (range)
Age of mothers (years old)		25 (5.87)	19 (17–36)
Mother’s education level	Junior high school	1 (11.1)	
	Senior high school	5 (55.6)	
	College/University	3 (33.3)	
Mother’s employment status	No	6 (66.7)	
	Yes	3 (33.3)	
Breastfeeding period	< 1 month	2 (22.2)	
	1–< 2 month	4 (44.4)	
	2–3 month	3 (33.3)	
Parity	Primiparous	5 (55.6)	
	Multiparous	4 (44.4)	
Household income (IDR)	< 1,500,000	2 (22.2)	
	1,500,000–3,000,000	4 (44.4)	
	> 3,000,000	3 (33.3)	
Marriage	Married	7 (77.8)	
	Unmarried	2 (22.2)	

**Table 2:** Themes identified through interviews with breastfeeding mothers

Themes
Strengthening elements
Maternal affection to her baby
Support system from family and community
Having adaptive coping strategy
Weakening elements
Impaired comfort
Insufficient milk supply
Financial problem
Parenting problem
Indifferent husband
